# Chemical and Electronic Structure Characterization of Electrochemically Deposited Nickel Tetraamino-phthalocyanine: A Step toward More Efficient Deposition Techniques for Organic Electronics Application

**DOI:** 10.1021/acs.jpcc.1c01396

**Published:** 2021-06-15

**Authors:** Maciej Krzywiecki, Sandra Pluczyk-Małek, Paulina Powroźnik, Czesław Ślusarczyk, Wirginia Król-Molenda, Szymon Smykała, Justyna Kurek, Paulina Koptoń, Mieczysław Łapkowski, Agata Blacha-Grzechnik

**Affiliations:** † Institute of Physics − CSE, Silesian University of Technology, Konarskiego 22B, 44-100 Gliwice, Poland; ‡ Faculty of Chemistry, Silesian University of Technology, Strzody 9, 44-100 Gliwice, Poland; § Faculty of Materials, Civil and Environmental Engineering, University of Bielsko-Biala, Willowa 2, 43-309 Bielsko-Biala, Poland; ∥ Institute of Engineering Materials and Biomaterials, Silesian University of Technology, Konarskiego 18A, 44-100 Gliwice, Poland; ⊥ Centre of Polymer and Carbon Materials, Polish Academy of Sciences, 34 Curie-Sklodowska Str., 41-819 Zabrze, Poland

## Abstract

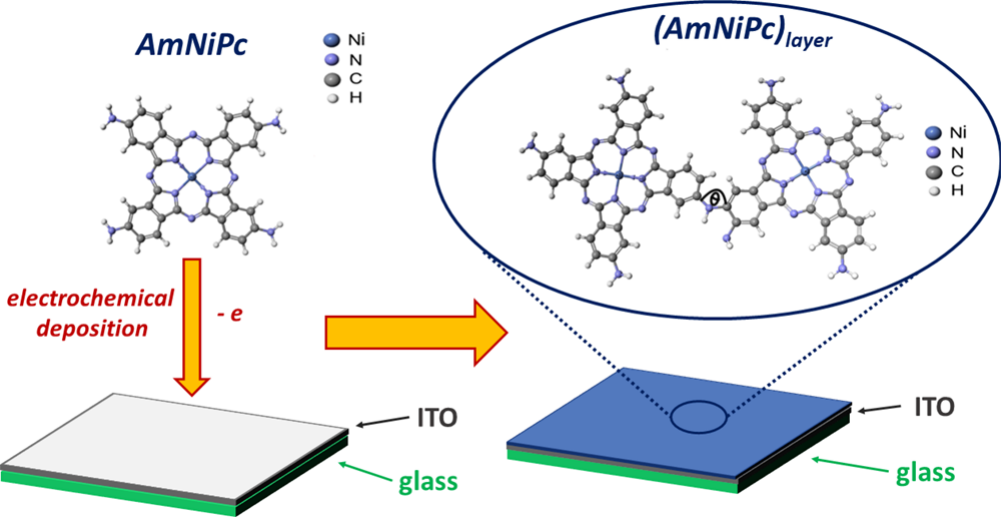

Phthalocyanines (Pc), with or without metal ligands, are still of high research interest, mainly for the application in organic electronics. Because of rather low solubility, Pc-based films are commonly deposited applying various advanced and demanding vacuum techniques, like physical vapor deposition (PVD). In this work, an alternative straightforward approach of NiPc layer formation is proposed in which NH_2_-side groups of nickel(II) tetraamino-phthalocyanine (AmNiPc) are engaged in the process of electrochemical deposition of (AmNiPc)_layer_ on indium-tin oxide (ITO) substrates. The resulting layer is widely investigated by cyclic voltammetry, atomic force microscopy, UV–vis, and ATR-IR spectroscopies, X-ray diffraction, and photoemission techniques: X-ray and UV-photoelectron spectroscopies. The chemical and electronic structure of (AmNiPc)_layer_ is characterized. It is shown that the electronic properties of the formed (AmNiPc)_layer_/ITO hybrid correspond to the ones previously reported for PVD-NiPc films.

## 1 Introduction

Phthalocyanines are conjugated macrocycles existing either in a free-base form (H_2_Pc) or with a metal in the center (MPcs). They have been widely studied for applications in (opto)electronic devices, such as solar cells,^1−3^ organic light-emitting diodes,^4,5^ gas sensors,^6−9^ and field-effect transistors.^10^ The big advantage of Pcs is that their macroscopic properties derive directly from their molecular electronic structure. Thus, the properties can be easily tuned by adding various substituent groups to the molecule or simply choosing different central metals. This, combined with easy processing and relatively high thermal and chemical stabilities,^11^ makes phthalocyanines attractive materials in the field of organic electronics. The biggest limitation of Pcs in some applications (i.e., solar cells, gas sensors) are low electron mobility and inefficient charge transport. These limitations can be overcome by combining Pcs with inorganic semiconductors in hybrid structures.^12^

Nickel phthalocyanine has been until now mainly investigated for the possible application not only in (opto)electronic devices, like gas sensors^6^ (e.g., ammonia^13−15^ toluene vapors,^16^ and nitrogen(II) oxide^17^), biosensors (e.g., dopamine^18^), solar cells,^19^ and supercapacitors^20^ but also in photocatalysis^21,22^ or photodynamic therapy.^23^

Metal-containing phthalocyanines are typically applied in the form of thin films. It has been shown that the selected deposition technique and/or process conditions can strongly influence the so called “supramolecular arrangement” of the resulting layer and thus its spectroscopic, electrical, or sensing properties.^24^ Various deposition techniques, like spin-coating, Langmuir–Blodgett, or layer-by-layer techniques can be applied for Pcs thin film formation. However, due to rather low solubility of Pcs, physical vapor deposition (PVD) remains the most common approach. Although vacuum techniques allow for almost ultimate control of the deposition parameters, which results in superior control of the layer properties, among others such as purity, morphology, and crystallinity,^25^ the disadvantage seems to be obvious—they are simply expensive.

Keeping in mind that one of the goals of technology nowadays is to seek for techniques resulting in an optimized cost-to-effect ratio, wet deposition seems to be an appealing alternative, since it fulfills this condition combining relatively easy and low-cost controlled layer production with the simultaneous sustainment of the acceptable layer quality in terms of the electronic structure and chemical and structural properties. One of the examples of the easy-accessible wet-deposition techniques is electrochemical polymerization. This electrodeposition method leads to direct growth of a synthesized material on the surface of a conductive substrate, which enables a straight-away use and in the meantime an unconventional approach for the characterization of electronic properties.^26,27^ Many papers concerning the electropolymerization of aniline, thiophene, pyrrole, or carbazole derivatives have been published until now, showing a wide range of possibilities of this method. It has been also reported that the Pc derivatives can be electrochemically deposited by the anodic oxidation of the outer substituents of the Pc ring.^28^

In this work, an alternative to vacuum-born NiPc films is shown. Here, we propose a deposition technology, which results in the stable organic layer of strictly defined properties not varying significantly from those obtained by expensive and well-established techniques. As a demonstrator, NiPc with four outer primary amine groups (AmNiPc, isomeric mixture) was selected as a precursor for the layer formation. The selection of the NiPc derivative was based on two premises: (i) the possibility of −NH_2_ units’ involvement in making NiPc suitable for electrochemical deposition and (ii) semiempirical computational modeling performed for verification if the electronic properties of NiPc were maintained on the course of the proposed deposition process. The chemical and electronic structure of the resulting layer formed on the indium-tin oxide (ITO) substrate was widely characterized by cyclic voltammetry (CV) and various spectroscopic techniques: UV–vis, attenuated total reflectance-infrared (ATR-IR), X-ray and UV-photoelectron spectroscopies (XPS and UPS, respectively), and the morphology was investigated by atomic force microscopy (AFM), while the crystalline structure by the X-ray diffraction (XRD) method.

## 2 Methods

### 2.1 Materials

Nickel(II) 2,9,16,23-tetra(amino)phthalocyanine (AmNiPc, isomeric mixture) was purchased from PorphyChem. Tetrabutylammonium tetrafluoroborate (TBABF_4_) (99%, Sigma-Aldrich) in dimethylformamide (DMF) (≥99.8%, Sigma-Aldrich) was used as an electrolyte solution for the electrochemical polymerization of AmNiPc and the electrochemical characterization of the resulting layer.

### 2.2 Computational Modeling

All theoretical simulations were performed using the SCIGRESS program (version FJ 2.8). The minimum energy geometries of the NiPc isolated molecule, AmNiPc monomer, and AmNiPc dimer have been found by the quantum chemistry PM6 method. The energies of molecular orbitals were calculated for the as-optimized geometries. The AmNiPc dimer was modeled in order to study the influence of AmNiPc layer formation on molecular orbital energy levels.

### 2.3 Electrochemical Deposition of Nickel(II) Tetraamino-phthalocyanine

The electrochemical deposition of nickel(II) tetraamino-phthalocyanine (AmNiPc) was done using a CHI 660C electrochemical workstation (CH Instruments Inc.). AmNiPc was dissolved in 0.1 M TBABF_4_/DMF electrolyte solution to form 0.1 mM solution. The resulting mixture was homogenized using ultrasonic mixing and then purged with argon (Ar) for 15 min before the electrochemical tests. The system consisted of three electrodes: a glassy carbon (GC, EDAQ, 1 mm dia.) or ITO/borosilicate glass electrode (Präzisions Glas & Optik GmbH, PGO) acting as a working electrode, Ag wire as a pseudoreference electrode, and a GC rod applied as a counter electrode. The electrodes were copiously rinsed with DMF and mounted in a Teflon holder prior to use. The electrochemical deposition of AmNiPc was conducted by means of CV with the following process parameters: the potential range: (−1.8, 1.4) V, the scan rate: 0.1 V/s, and 3, 5, 10, or 15 scan cycles. The further investigations were conducted on the layer obtained with 10 scan cycles (unless stated otherwise). Ferrocene (Fc/Fc^+^) was used as a reference for the potential calibration.

### 2.4 Investigation of the Morphology and Chemical, Crystalline, and Electronic Structure of Electrochemically Deposited (AmNiPc)_layer_

Morphology of the layers was checked with a PSIA XE-70 atomic force microscope working in a noncontact mode. The monolithic silicon probes TAP-300AL-G were used in diamond—like coating variant (resonance frequency 300 kHz, spring constant 40 Nm^–1^, as delivered by BudgetSensors) in order to reduce the surface water meniscus—related interactions. The images were processed with use of Gwyddion SPM software to correct sample inclination and distortions caused by the z-scanning stage.^29^ No other corrections to the images were made.

The electrochemical properties of the deposited layer were investigated in pure electrolyte solution,0.1 M TBABF_4_/DMF, using a CHI 660C electrochemical workstation (CH Instruments Inc.) and the abovementioned conventional three-electrode system with GC or ITO covered with (AmNiPc)_layer_ acting as a working electrode. The electrolyte solution was bubbled with Ar for 15 min prior to tests. The CV measurements were done within the (−1.8, 1.2) V potential range with the scan rate equal to 0.1 V/s.

UV–vis spectra of ITO covered with (AmNiPc)_layer_ and 0.05 mM solution of AmNiPc in DMF were recorded with a Hewlett Packard 8452A UV–vis spectrometer.

IR spectra of the (AmNiPc)_layer_ deposited on ITO and powder AmNiPc were collected in the ATR mode on a PerkinElmer IR spectrometer.

XRD, in the angle range from 5 to 45°, was carried out with a URD-65 Seifert (Germany) diffractometer in a Bragg–Brentano geometry. CuKα radiation was used at 40 kV and 30 mA. Monochromatization of the beam was obtained by means of a nickel filter and a graphite crystal monochromator placed in the diffracted beam path. A scintillation counter was used as a detector.

The photoemission experiment was conducted at a multichamber ultrahigh-vacuum experimental setup (base pressure 7 × 10^–11^ mbar) equipped with a PREVAC EA15 electron energy analyzer. For XPS purposes, the sample was excited with Al Kα radiation (1486.6 eV, PREVAC XR40B1 source). The spectra were recorded with a normal take-off angle and the analyzer’s binding energy scale was calibrated to Au 4f_7/2_ (84 eV^30^). For survey spectra, the pass energy (PE) was set to 200 eV while for high-resolution scans of particular energy regions, the PE 100 eV was applied. The spectra underwent decomposition with use of CASA XPS software, its integrated algorithms, and sensitivity factors.^31^ For the component representation, the sum of Gauss (30%) and Lorentz (70%) line shapes were used and the Shirley-type background was applied. The full width at half maximum (FWHM) of particular components was allowed to vary within a narrow range in order to optimize the fitting residue. For UPS examination, the same experimental setup was used but the sample was irradiated with a He plasma discharge source (PREVAC UVS40A2) giving a He I line with excitation energy of 21.22 eV. The PE was set to 5 eV and the scanning step to 5 meV.

## 3 Results and Discussion

### 3.1 Computational Modeling

**Figure 1:**
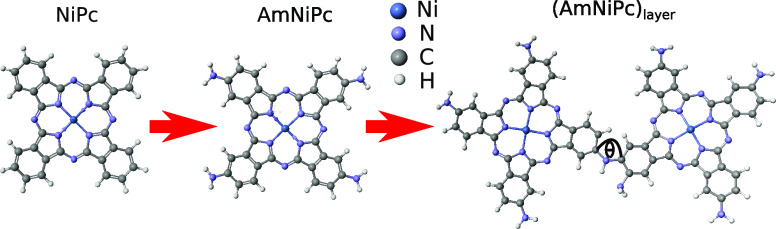
Chemical structure energy global minimum geometries of NiPC, AmNiPc monomer, and (AmPc)_layer_ investigated in this work.

In order to predict the changes in the electronic properties caused by NiPc modification and (AmNiPc)_layer_ formation, semiempirical calculations were performed. The energy levels of the highest occupied molecular orbital (HOMO) and lowest unoccupied molecular orbital (LUMO) were calculated for NiPc and AmNiPc monomers as well as for the AmNiPc layer represented by the AmNiPc dimer (Figure 1). As we expect that −NH_2_ groups are involved in the formation of the layer in the electropolymerization process, we optimized the geometry of a dimer made of two AmNiPc molecules joined through the −NH_2_ unit. The geometry was optimized for the bond angle between two molecules (Figure 1). The global energy minimum was obtained for θ = 131°.

The absolute values of HOMO and LUMO levels as well as the band gap (*E*_g_) cannot be compared with the experimental ones, since semiempirical methods based on Hartree-Fock theory (e.g., PM6 method applied here) overestimate the *E*_g_ values.^32^ However, the changes in the energy level obtained from modeling can be considered reliable. Thus, from the changes in HOMO and LUMO levels, we can estimate the expected changes in *E*_g_, ionization energy (IE), and electron affinity (EA) between the NiPc and AmNiPc monomer as well as the AmNiPc monomer and (AmNiPc)_layer_ (Table 1). From Table 1, one can see that addition of amine groups to NiPc causes decrease in *E*_g_ and IE and increase in EA, but the changes are relatively low. The formation of (AmNiPc)_layer_ does not introduce further changes in IE but cause a decrease in the EA value. As based on the theoretical modeling, we do not expect significant changes in electronic properties; we select AmNiPc as a precursor for layer formation.

**Table 1:** Values of *E*_g_, IE, and EA Shift between NiPc and AmNiPc as well as AmNiPc and (AmNiPc)_layer_ Calculated by the PM6 Semiempirical Method

	AmNiPc–NiPc	(AmNiPc)_layer_–AmNiPc
Δ*E*_g_, eV	–0.2	–0.1
ΔIE, eV	–0.35	0
ΔEA, eV	0.16	–0.13

### 3.2 Electrochemical Characterization of AmNiPc and Electrodeposition of (AmNiPc)_layer_

**Figure 2:**
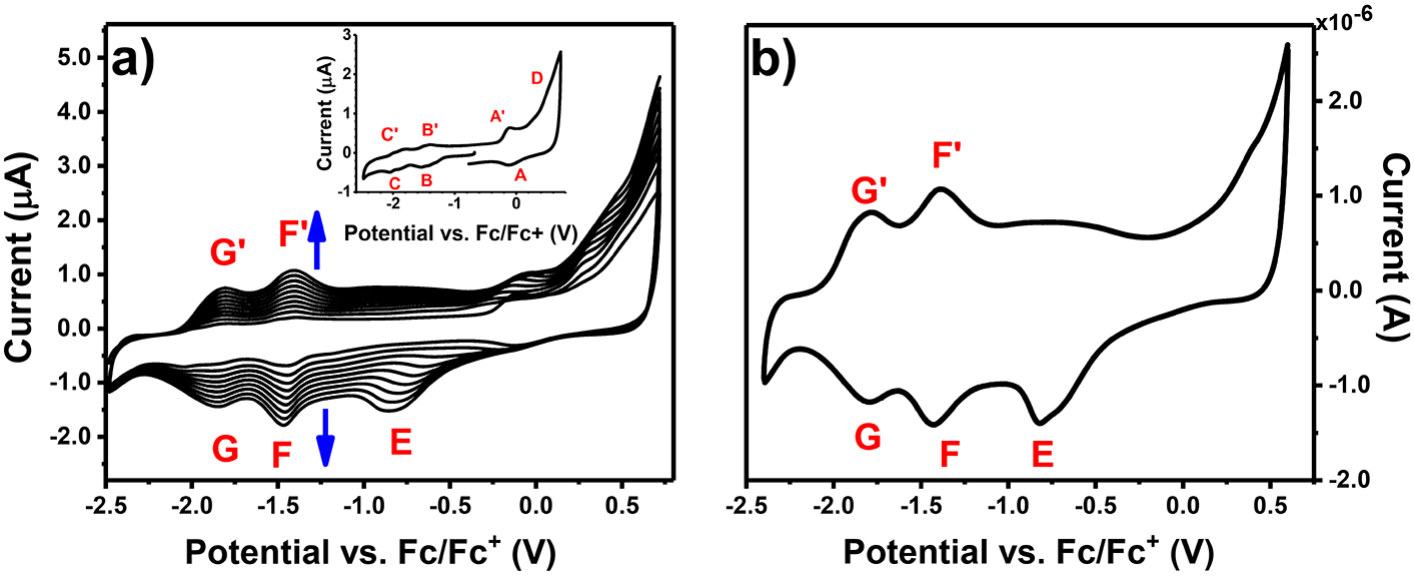
(a) CV curves recorded in 0.1 mM AmNiPc electrolyte solution (0.1 M TBABF_4_/DMF) with GC as a working electrode; inset: the first scan (b) CV curves recorded in 0.1 M TBABF_4_/DMF electrolyte solution with (AmNiPc)_layer_/GC (10 cycles) as a working electrode.

Figure 2a presents the CV curves recorded during continuous scanning in the AmNiPc solution. In the first cycle (Figure 2a inset), the characteristic redox couples of the Ni-containing phthalocyanine ring are observed at ca. −0.1 V (A/A′), −1.5 V (B/B′), and −1.9 V (C/C′) and correspond to one-electron processes occurring in the Pc ring, namely, [NiPc^2–^]/[NiPc^1–^]^1+^, [NiPc^2–^]/[NiPc^3–^]^1–^, and [NiPc^3–^]^1–^/[NiPc^4–^]^2–^, respectively.^33−36^ Based on the first oxidation and reduction peak, the ionization potential (IP) and EA were estimated (Table 2). In accordance with Koopmans’ Theorem, these values can be correlated with the energy values of the HOMO and LUMO, respectively, which also enables the HOMO–LUMO gap (band gap) calculation. Using the −4.8 eV value for the Fc standard with respect to the zero-vacuum level, the following values were obtained: IP = 4.69, EA = −3.26, and electrochemical band gap: *E*_g_el__ = 1.43 eV, which is comparable with the values obtained for similar systems.^37,38^

**Table 2:** Thickness of (AmNiPc)_layer_ Deposited on ITO with Various Numbers of Electrodeposition Cycles, Estimated with AFM

	number of cycles of electrodeposition			
	3 cycles	5 cycles	10 cycles	15 cycles
thickness of (AmNiPc)_layer_/ITO (nm)	70	110	150	180

In the first anodic scan the irreversible oxidation of AmNiPc can be observed at ca. 0.75 V (Figure 2a inset), which can be assigned to the oxidation of the outer primary amine groups resulting in the formation of the radical cation. During the continuous scanning in the broad potential range, the increase in the registered current can be observed, indicating the successive deposition of the electroactive layer on the working electrode surface. The new, rising signals (E/E′, F/F′, and G/G′) correspond to the electrochemical activity of the obtained layer. The suggested mechanism of the electrodeposition process consists of the oxidation of the primary amine group, as in the case of the electrochemical polymerization of aniline.^28^ The steady increase in the recorded current occurred up to 15 scan cycles. Above this number, the rebuilding of the redox couples was observed, together with the slow decrease in the recorded current, suggesting the progressive degradation of the layer.

### 3.3 Morphology and Chemical and Electronic Structure of Electrochemically Deposited (AmNiPc)_layer_

**Figure 3:**
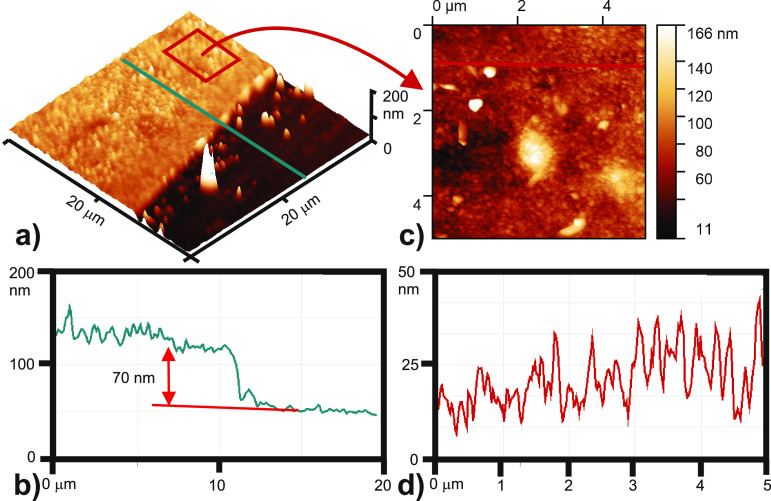
(a) AFM scan image (20 × 20 μm^2^) of the AmNiPc layer edge (b) extracted from line in the panel a cross-section profile; (c) 5 × 5 μm^2^ magnification of the AmNiPc surface with the respective (marked with red line) cross-section profile (d).

The electrochemically deposited (AmNiPc)_layer_ was investigated in the next step by means of microscopy, electrochemical, spectroscopic, and photoemission techniques. First, the (AmNiPc)_layer_/ITO layer integrity was checked by topography investigations made with AFM examination. The investigations confirmed that the organic films are uniformly covering the substrates without any discontinuities within the layers, as proved not only by AFM scans, but also by optical microscope coupled with AFM. The additional purpose of these studies was to validate the deposited film thickness based on the substrate-AmNiPc layer edge height. Figure 3a shows the exemplary 20 × 20 μm^2^ image of the layer edge obtained for the three-cycle AmNiPc film. The AFM scan shows a uniform surface consisting of densely packed crystallites of rather vertical orientation. The greenish line crossing the edge indicates the profile extraction area which is presented in panel b. Although some distortions can be noticed (e.g., residual crystallites protruding from the ITO substrate uncovered by AmNiPc), the film thickness can be estimated as ca. 70 nm. The procedure was repeated for the set of layers obtained with the higher number of deposition cycles, i.e., 5, 10, and 15 (Table 2). As expected, the relation between the number of the electrodeposition cycles and the film thickness is not linear. This indicates that at a certain point of the deposition process, the further buildup of the electroactive species on the electrode surface makes the resulting film more densely packed rather than significantly thicker.

Figure 3c presents 5 × 5 μm^2^ magnification of the film surface while panel d shows the cross-section made in the area marked with the red line. The rough analysis of the image and the cross-section profile confirms rather vertical orientation of the AmNiPc crystallites with a diameter in the range of tens of nm. Moreover, at some areas of the AFM image the bigger crystallites protruding from the layer are also visible. Both findings are consistent with previous studies made on similar phthalocyanine structures (like FePc and CuPc) and of a similar order of thickness range.^39,40^

The CV curves registered for the GC working electrode covered with (AmNiPc)_layer_ in the monomer-free electrolyte solution is shown in Figure 2b. Electrochemical reduction of (AmNiPc)_layer_ proceeds as a two-step process, which is observed by two redox couples: F/F′ and G/G′. The first reduction step occurs at −1.42 V (F) and the second one at −1.80 V (G). In the anodic cycle, oxidation of reduced forms is registered at −1.78 V and −1.39, which indicated that both reduction steps are reversible. Additionally, in the wide range of potentials, the electrochemically irreversible oxidation of the obtained layer is registered. The signal in the anodic cycle is without a clearly defined maximum; however, in the cathodic cycle, the reduction of oxidized layers can be evidently seen at −0.82 V (E). As in the case of AmNiPc, based on the potentials of the first reduction peak, the EA of (AmNiPc)_layer_ was calculated (Table 3). Electrodeposition leads a product obtained with a bit lower EA (−3.38 eV), the observed changed comparing to monomer AmNiPc is in agreement with computational modeling data (Table 1). Due to not clear signal of oxidation, the IP of the deposited layer was not calculated and thus the electrochemical band gap as well.

**Table 3:** Electrochemical Data

	*E*_red_ (V)	*E*_ox_ (V)	EA^a^ (eV)	IP^b^ (eV)	*E*g_el_^c^ (eV)
AmNiPc	–1.54	–0.11	–3.26	4.69	1.43
(AmNiPc)_layer_	–1.42		–3.38		

a Electron affinity: EA = −(*E*_red_ + 4.8)/eV.

b Ionization potential: IP = *E*_ox_ + 4.8/eV

c Electrochemical band gap: *E*_g_el__ = | – IP – EA|/eV.

**Figure 4:**
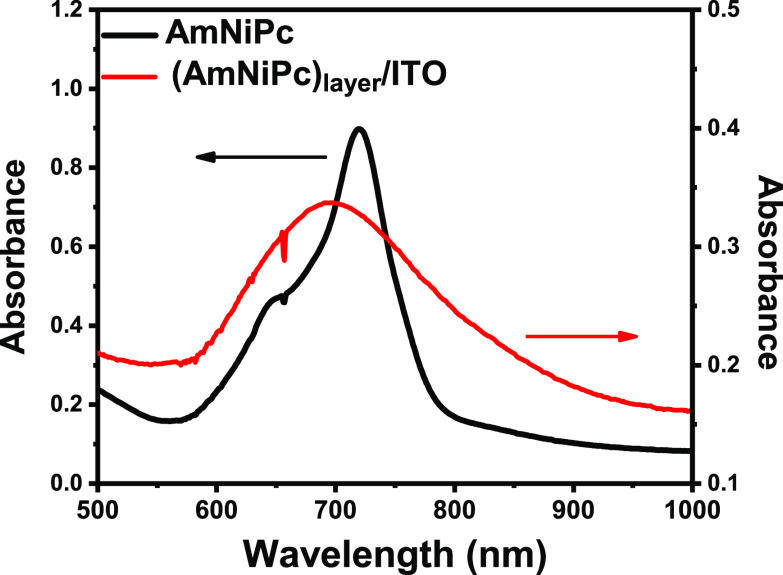
UV–vis spectra of 0.1 mM AmNiPc in DMF (black line) and (AmNiPc)_layer_/ITO (red line).

Figure 4 shows the UV–vis spectra of AmNiPc in the solution and in the form of a layer deposited on the ITO electrode. In solution, the Q band of AmNiPc (π → π* transition) is observed at 720 nm.^33,41^ The sharp and singlet Q band is typical for monomeric Pc that does not undergo aggregation. As this represents π → π* transition, the edge of this band can be used for determination of the optical band gap which is equal to 1.59 eV (Table 4). This value is slightly larger than that estimated from CV measurement but is in agreement with the literature data for similar compounds.^38^ The Q band of (AmNiPc)_layer_ is significantly broadened, possibly due to aggregation.^33,38^ Hence, the Q band edge of the layer is bathochromically shifted as compared with the solution of AmNiPc and corresponds to an optical band gap of 1.40 eV.

**Table 4:** Optical Data

	λ_edge_ (nm)	*E*_g_opt__(eV)^a^
AmNiPc	780	1.59
(AmNiPc)_layer_	882	1.40

a Optical band gap: *E*g_opt_ = 1240/λ_edge_.

**Figure 5:**
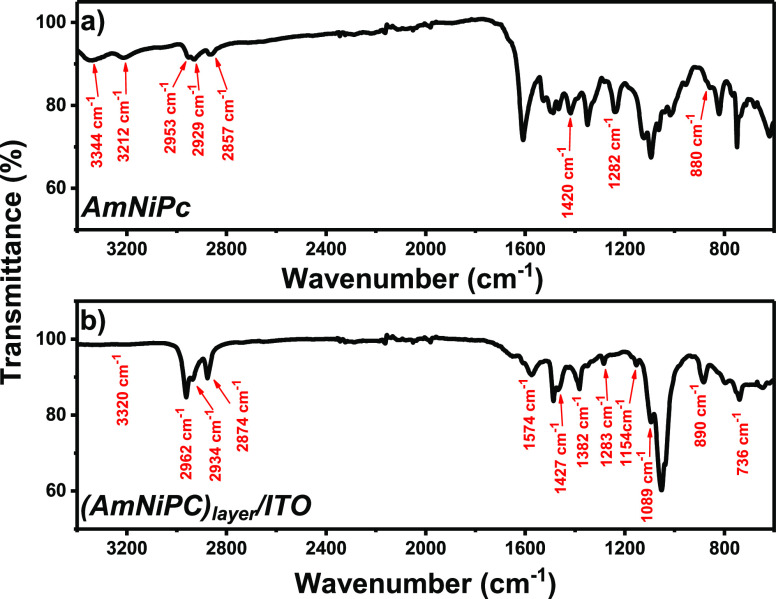
ATR-IR spectra of AmNiPc (black line) and (AmNiPc)_layer_/ITO (red line).

The ATR-IR spectra collected for AmNiPc and the resulting (AmNiPc)_layer_ are presented in Figure 5. The complete assignment of the registered signals is given in Table S1. In both spectra, the characteristic bands of the phthalocyanine ring are observed at 2958 ± 6, 2866 ± 9, and 2900 ± 3 cm^–1^ that arise from the stretching of C–H bonds in the ring and the C–H (sp^3^) symmetric and asymmetric stretching vibrations, respectively.^42^ The bands at 1282 ± 1 and 1423 ± 4 cm^–1^ can be assigned to the C–N and C–C bond stretching in isoindole units.^43^ Moreover, the vibrations of the nickel ligand are observed at 880 and 890 cm^–1^ for powder and electrodeposited AmNiPc, respectively.^43^ Since no band characteristic for N–H bending vibrations of unsubstituted Pc is registered at ca. 1000 cm^–1^,^42,44,45^ the purity of both the powder and formed layer is verified.

The presence of the outer primary amine groups in AmNiPc are confirmed by the stretching vibrations of N–H observed at 3344 and 3212 cm^–1^.^46^ They are significantly suppressed for (AmNiPc)_layer_, thus suggesting the involvement of −NH_2_ groups in the formation of the layer. The broad band at 3320 cm^–1^ registered for (AmNiPc)_layer_/ITO can be assigned to N–H stretching in both unreacted primary amines and the secondary amino groups formed in the electrochemical process. This is further confirmed by the appearance of the band at 1382 cm^–1^ in the IR spectrum of the deposited layer, which can be assigned to the stretching vibrations of C–N= located between benzenoid and quinoid units and the 1154 cm^–1^ mode of Q = NH^+^–B characteristic for the polyaniline-like structure.^47−49^ The abovementioned observations are in agreement with the previous report on the mechanism of electropolymerization of aminoPc.^28^

**Figure 6:**
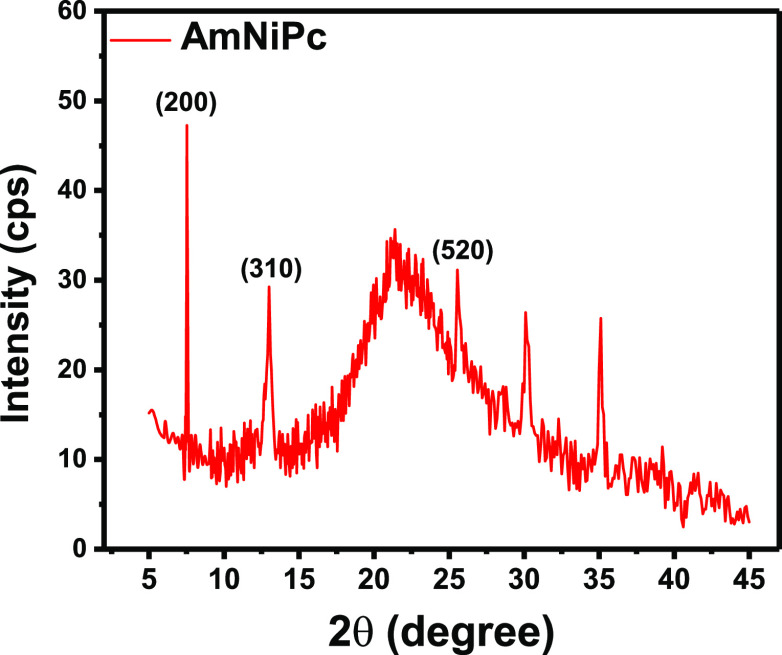
XRD pattern of the AmNiPc layer deposited on ITO.

Finally, since the 700–800 cm^–1^ region of the IR spectrum of phthalocyanines can be used for the identification of their crystal packing arrangement,^43,44,50^ the crystallographic orientation of (AmNiPc)_layer_/ITO was analyzed. The out-of-plane C–H bending vibrations occurring at 736 cm^–1^ and the absence of the band at ca. 780 cm^–1^ in the recorded spectrum suggest the presence of the α-phase in the film.^51^ This is in agreement with the XRD pattern of the AmNiPc layer deposited on ITO (Figure 6) in which a strong reflection peak is observed at 2θ ≈ 7° that can be assigned to the reflection from the (200) crystalline plane of α-phase NiPc.^51,52^ The other peaks, observed at 2θ ≈ 13^°^ and 2θ ≈ 24.5°, represent (310) and (520) reflections of the tetragonal structure. A broad halo centered at 2θ = 22^°^ as well as peaks observed at 2θ > 30° are caused by scattering from the ITO substrate. Using the familiar Scherrer’s formula, the size of AmNiPc crystallites was calculated yielding a value of 65 nm. This value is in good agreement with the values reported for other nickel phthalocyanine thin films.^53,54^

**Figure 7:**
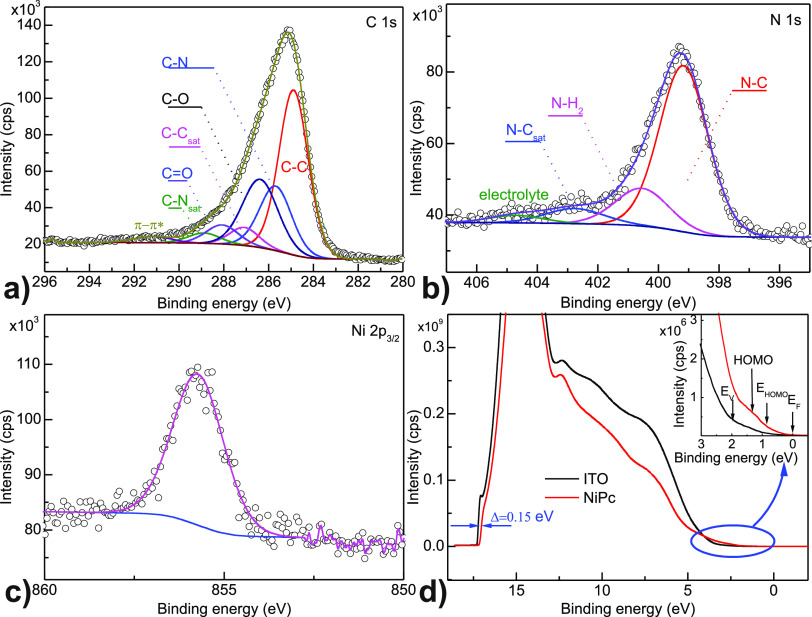
(a–c) High-resolution spectra of C 1s, N 1s, and Ni 2p_3/2_ XPS energy regions decomposed into main components; (d) comparison of UPS taken for the ITO substrate (black) and ITO covered with the AmNiPc layer (red); the inset in panel d presents magnification of the VB/HOMO energy region. For details see text.

Following the ATR-IR and XRD examinations, the photoemission experiment was conducted. The summary of the XPS experiment is presented in Figure 7. Panel a presents the C 1s region decomposed into main AmNiPc layer-originating components (confirmed also above by ATR-IR findings), i.e., the C–C/C–H component at 284.8 eV together with its satellite feature at ∼286 eV.^55^ Next, the C–N component at ∼285.8 eV with its satellite feature (∼288.9 eV) and barely detectable π–π* shake-up at ∼292.0 eV can be observed. The classical expected phthalocyanines three-branch spectrum^55,56^ of C 1s is somehow affected by substrate-and ambience-originating intrusion as manifested by C–O/C=O components. Moreover, the C–N satellite seems to be affected by the C–OH/COOH groups since its intensity is bigger than expected on the basis of similar compound analysis. The intrusion existence is supported with the O 1s spectrum (see Supporting Information, Figure SI.1) which clearly points the existence of oxygen-related carbonaceous contaminations together with the residual impact of substrate components. Following C 1s, the N 1s spectrum is shown in Figure 7b. The region was decomposed into N–C (399.0 eV, this component due to its increased FWHM is also responsible for the C–N–Ni configuration) with its satellite feature at 402.7 eV.^55^ The deviation from the classic phthalocyanine-like spectrum is the NH_2_ component (corresponding to the C–N–H_2_ configuration) at ∼400.5 eV and the barely traceable electrolyte-related component. Based on the ratio between the area of N–C and N–H components, it can be estimated that ca. 1.5 NH_2_ groups per AmNiPc molecule are involved in the formation of the AmNiPc layer; thus, dimer and trimers are mainly formed. This is in agreement with, e.g., UV–vis results in which only a slight shift in the optical band gap was observed.

The last aspect is the Ni 2p check to examine the chemical integrity of the AmNiPc layer. Only one component is detectable representing Ni–N bonding. Although the component is relatively wide (FWHM is slightly below 2 eV), it is highly symmetrical and no another component’s existence is detectable. The peak’s broadening most likely is due to the significant disorder of the AmNiPc layer.

For the electronic structure photoemission-based examination, the UPS method was used, and the results are shown in Figure 7d. In order to assure that the substrate is not influencing the energy parameter determination of the AmNiPc layer, the ITO-derived spectrum was shown in Figure 7d overlapped with the AmNiPc-originating one (black line stands for ITO and red line for AmNiPc, respectively). The clear shift of the high-energy cutoff can be seen (of a value of 0.15 eV) toward higher kinetic energy (for the AmNiPc layer). Furthermore, the decrease in photoemission signal intensity is visible in the midbinding energy region as a result of attenuation of substrate-related photoelectrons by the organic overlayer exhibiting lower density of states (DOS). Next, in the valence band (VB) region of the spectra, the situation is being inversed (see inset to Figure 7d). In the region above the ITO’s VB (namely, in its band gap region), the additional DOS appears which can be related to AmNiPc -related molecular orbitals. The weak signal originating from the HOMO can be detected at 1.26 eV binding energy (BE). Next, following the assumption that the Fermi level (*E*_F_) for the analyzer and investigated sample are equal, it was possible to determine the energy difference between the *E*_F_ and the HOMO onset *E*_F_–*E*_HOMO_, the work function ϕ, and IE.

The surface work function of the examined AmNiPc was determined according to φ *= hv* – *E*_cutoff_; where *hv* is the excitation energy (here, 21.22 eV) and *E*_cutoff_ is the interception point of the high BE cutoff of the photoemission spectrum.^57^

Next, the ionization energy was determined as IE *=* φ + (*E*_F_*– E*_HOMO_).^57^ Determining the *E*_F_–E_HOMO_ as 0.55 eV, the determined electronic parameters were φ = 4.08 eV, IE = 4.63 eV (with an uncertainty of 0.07 and 0.09 eV, respectively). The data are, to a certain accuracy, in agreement with the ones obtained for NiPc deposited onto gold, silver, and ITO substrates^32,53,58,59^ and, which is probably more important, is close to 4.69 eV of IP resulting from electrochemical characterization for isolated AmNiPc molecules. The slight deviation is however expected since AmNiPc layers are of slightly different composition (existence of amine groups at the molecule corners)—hence different molecule–molecule interactions extorting different (to some extent) molecular orbital overlaps, and as a matter of fact, the latter strictly defines the electronic parameters of the organic layers.

Using the *E*_g_ value determined by means of the UV–vis experiment (i.e., *E*_g_ = 1.40 eV—see previous sections) for the AmNiPc layer, the electron affinity may be also determined as EA *=* IE – *E*_g_. Of course, the data shall not be considered as arbitrary comparable, since the optical *E*_g_ is most likely slightly smaller than transport *E*_g_ for similar systems.^60−63^

As a result, the EA = 3.23 eV which is almost a perfect match with electrochemistry. Some deviations are, however, expected here since electrochemical and photoemission experiments differ environmentally. What is also quite crucial for the purposes of the presented studies is not only the similarities between the values obtained on the course of different methods but also the fact that the shift of the IE value between NiPc and AmNiPc (0.35 eV; as shown by computations) is consistent with the difference between IE obtained from UPS for (AmNiPc)_layer_ and the values of IE reported in the literature for NiPc (0.3–0.4 eV).^58,59^

## 4 Conclusions

In the presented work, amino-substituted nickel phthalocyanine has been electrochemically deposited on the ITO surface. The morphological, chemical, crystalline, and electronic structure of the resulting layer was widely characterized using electrochemical, microscopy, spectroscopic, XRD, and photoemission techniques. The obtained data indicate that the deposition process occurs via outer primary amino groups and aniline-like electropolymerization mechanism. It has been shown that the electronic properties of the (AmNiPc)_layer_/ITO system are consistent with the NiPc layers obtained using the PVD technique, including the film structure, crystallinity, and (to some extent) thickness control. Hence, the presented solution-based low-cost approach may be an attractive alternative to highly demanding high-vacuum deposition techniques for formation of materials for organic electronics applications.

## Supporting Information Available

Assignment of IR signals recorded for AmNiPc and (AmNiPc)_layer_ (Table.SI.1) and O 1s high-resolution energy region recorded for (AmNiPc)_layer_ (Figure.SI.1) (PDF)
