# Supplementary material for: Chemical and Electronic Structure Characterization of Electrochemically Deposited Nickel Tetraamino-phthalocyanine: A Step toward More Efficient Deposition Techniques for Organic Electronics Application
Source: J Phys Chem C Nanomater Interfaces. 2021 Jun 15;125(24):13542–50. doi: 10.1021/acs.jpcc.1c01396 (PMC8282193; doi:10.1021/acs.jpcc.1c01396)
Supplement: Supplementary file 1 — jp1c01396_si_001.pdf [file jp1c01396_si_001.pdf]

# Supporting Information

for

# Chemical and Electronic Structure Characterization of Electrochemically Deposited Nickel Tetraamino-phthalocyanine: a Step Toward More Efficient Deposition Techniques for Organic Electronics Application

Maciej Krzywiecki [a*], Sandra Pluczyk-Małek [b*], Paulina Powroźnik [a], Czesław Ślusarczyk [c], Wirginia Król-Molenda [b], Szymon Smykała [d], Justyna Kurek [b], Paulina Koptoń [b], Mieczysław Łapkowski [b,e] and Agata Blacha-Grzechnik [b†]

[a] Institute of Physics – CSE, Silesian University of Technology, Konarskiego 22B, 44-100 Gliwice, Poland
[b] Faculty of Chemistry, Silesian University of Technology, Strzody 9, 44-100 Gliwice, Poland
[c] Faculty of Materials, Civil and Environmental Engineering, University of Bielsko-Biala, Willowa 2, 43-309 Bielsko-Biala, Poland
[d] Institute of Engineering Materials and Biomaterials, Silesian University of Technology, Konarskiego 18A, 44-100, Gliwice, Poland
[e] Centre of Polymer and Carbon Materials, Polish Academy of Sciences, 34 Curie-Sklodowska Str., 41-819 Zabrze, Poland

* Authors had equal contribution to this manuscript.
† Corresponding author:
E-mail: agata.blacha@polsl.pl
phone: +48 322371024
fax: +48 322371509
Address: 44-100 Gliwice, Strzody 9, Poland

*Tab.SI.1. Assignment of IR signals recorded for AmNiPc and $(AmNiPc)_{layer}$*

| **AmNiPc** *wavenumber ($cm^{-1}$)* | **(AmNiPc)layer/ITO** *wavenumber ($cm^{-1}$)* | **Assignment** |
|---|---|---|
| 3344 | | N-H stretching in primary amines |
| | 3320 | N-H stretching in primary and secondary amines |
| 3212 | | N-H stretching primary amines |
| 2953 | 2962 | $sp^2$ C-H stretching (ring) |
| 2929 | 2934 | symmetric $sp^3$ C-H stretching (alkyl) |
| 2857 | 2874 | symmetric $sp^3$ C-H stretching (alkyl) |
| 1612 | 1612 | stretching C-C in pyrrole |
| | 1574 | Quinoid ring stretching |

| | | |
|---|---|---|
| 1527 | | Bending C-H aryl |
| 1495 | | C-H bending |
| 1467 | 1466 | C–H in-plane bending |
| 1420 | 1427 | C-C and C-N asymmetric stretching in isoindole |
| | 1382 | C–N stretching in Q-B |
| 1345 | 1351 | C-C stretching in isoindole |
| 1282 | 1283 | C–N stretching in isoindole |
| | 1154 | In-plane C-H bending in Q=NH+–B |
| 1124 | 1113 | C–H in-plane bending |
| 1096 | 1089 | C–H in-plane bending |
| 1061 | 1048 | C–H in-plane deformation + C-N stretching in pyrroles |
| 880 | 890 | Ni-N stretching |
| 818 | | isoindole breathing + Ni-N stretching |
| 744 | | Out-of-plane C-H bending |
| 731 | 736 | Out-of-plane C-H bending |

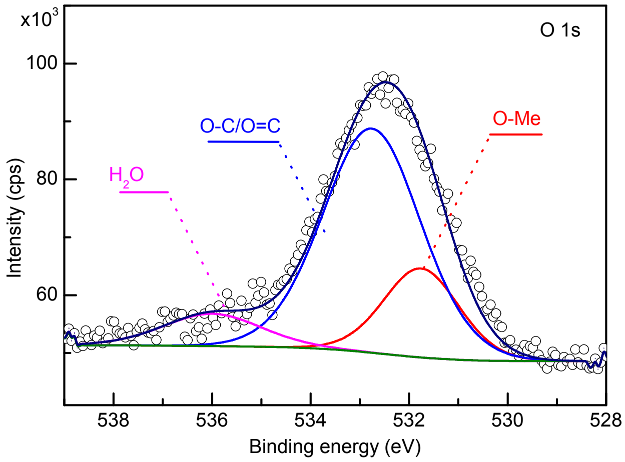


*Fig.SI.1 O 1s high – resolution energy region recorded for (AmNiPc)$_{layer}$*

The decomposed high – resolution O 1s energy region recorded with XPS experiment is presented in Fig.SI.1. besides substrate – related oxygen - metal component at ~531.5 eV, the

ambience originating carbonaceous adsorbates’ contribution can be noticed. At high binding energy side the trace of the adsorbed water vapor component is also detectable.
